# Supplementary material for: Delineation of complex gene expression patterns in single cell RNA-seq data with ICARUS v2.0
Source: NAR Genom Bioinform. 2023 Mar 29;5(2):lqad032. doi: 10.1093/nargab/lqad032 (PMC10052380; doi:10.1093/nargab/lqad032)
Supplement: lqad032_Supplemental_Files [file lqad032_supplemental_files.zip › Supplementary_Table1_Legend.docx]

**Supplementary Table 1 Top genes that change as a function of pseudotime.** Trajectory analysis was undertaken using the Monocle3 algorithm. The Moran’s I statistic is a measure of a gene’s spatial autocorrelation in a focal region of high dimensional space (i.e., cell clusters in UMAP space). Moran’s I value ranges from -1 to +1. A value of +1 indicates that gene expression is localised to a particular area in high dimensional space and expressed differentially across clusters.
