# Supplementary material for: Delineation of complex gene expression patterns in single cell RNA-seq data with ICARUS v2.0
Source: NAR Genom Bioinform. 2023 Mar 29;5(2):lqad032. doi: 10.1093/nargab/lqad032 (PMC10052380; doi:10.1093/nargab/lqad032)
Supplement: lqad032_Supplemental_Files [file lqad032_supplemental_files.zip › Supplmentary_Figure1.docx]

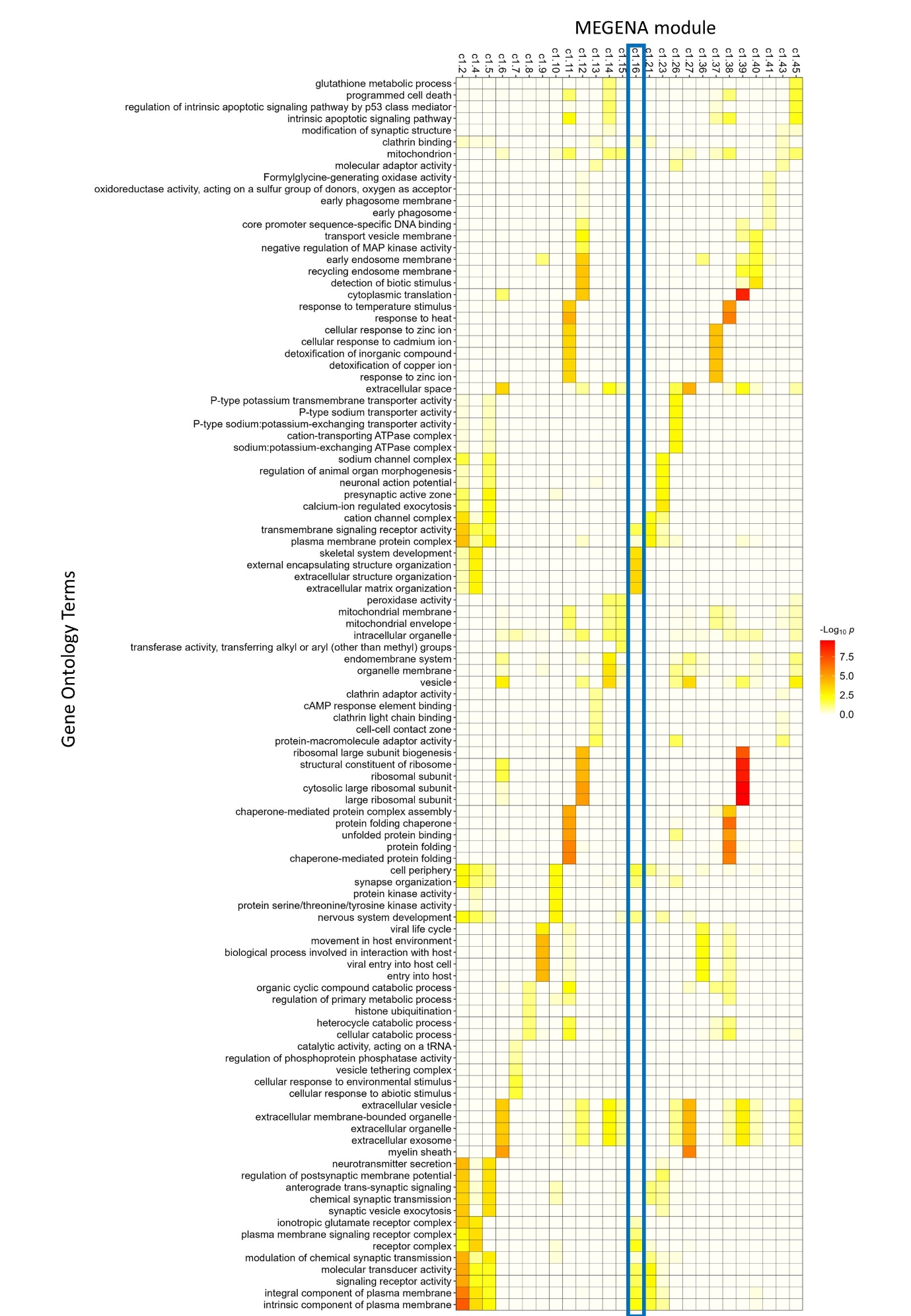


**Supplementary 1** Gene Ontology terms for MEGENA computed gene modules. Module c1_16 (highlighted in blue) involved in extracellular organisation, synapse signalling and developmental morphogenesis was predominately expressed in oligodendrocyte precursor cells.
